# Advances Postharvest Preservation Technology

**DOI:** 10.3390/foods12081664

**Published:** 2023-04-17

**Authors:** Maria Cefola, Bernardo Pace

**Affiliations:** Institute of Sciences of Food Production, National Research Council of Italy (CNR), c/o CS-DAT, Via Michele Protano, 71121 Foggia, Italy; maria.cefola@sipa.cnr.it

Fruits and vegetables are important sources of nutrients such as vitamins, minerals, and bioactive compounds, which provide many health benefits. However, due to suboptimal postharvest management, large quantities of fresh or fresh-cut fruit and vegetables lose their quality and nutritional value before they reach the consumer.

In this Special Issue, *Advances in Post-harvest Preservation Technology*, belonging to the Section “Food Packaging and Preservation”, 14 research and 2 review articles are included. Five research topics are addressed: (i) storage and treatment; (ii) packaging and edible coating; (iii) techniques or markers for determining fruit quality; (iv) prediction models; and (v) postharvest technologies and preservation strategies.

Pertaining to the first topic, the article published by Fatchurrahman et al. [1] reports the quality change of fresh goji berry fruit (*Lycium barbarum* L.) stored at different temperatures (0, 5, and 7 °C) for up to 12 d, highlighting significant differences concerning the fruit’s phytochemical attributes (vitamin C, soluble solid content, titratable acidity, total polyphenol, 2,2-diphenylpicrylhydrazy (DPPH), and anthocyanin content). This study revealed that goji fruit is optimally stored at a temperature of 5 °C for 9 d. At this temperature, physiological disorders (such as pitting and shriveling) were reduced, while the overall sensorial and nutritional quality attributes, including vitamin C levels, total soluble solid content, and antioxidant activity, were preserved. However, additional technologies such as modified atmosphere packaging may be required to better control decay and extend shelf life. Ma et al. [2] analyzed different forms of walnuts (*Juglans regia* L. cv. Xiangling, with a green husk, shell, and fresh kernels) stored between 0 and −20 °C for 10 months. The results showed that the walnuts with a green husk presented higher-quality kernels compared to the other forms of walnuts analyzed when stored at 0 °C for 3 months. Whereas at a freezing temperature (−20 °C) and during long storage, the walnuts with shells showed advantages with respect to maintaining fatty acid content, total phenols, and total ascorbic acid concentrations compared with other forms of walnuts. To preserve the quality of fresh-cut cauliflower (*Brassica oleracea* L. *botrytis* cv. Xuebai), vegetables were immersed in a solution containing calcium chloride (CaCl_2_) at a 2% concentration and at different temperatures (0, 20, or 40 °C) for 10 min [3]. While stored at 4 °C for 15 d, changes in sensory quality, firmness, color, ascorbic acid concentration, and the total glucosinolates, polygalacturonase, and lipoxygenase concentrations of cauliflower florets were evaluated. The results showed that a treatment with CaCl_2_ and high temperature (40 °C) maintained a higher firmness value, preventing the browning and yellowing of fresh-cut cauliflower florets. This treatment was optimal for preventing reductions in ascorbic acid content and total glucosinolates and inhibiting the activity of polygalacturonase and the softening of cauliflower florets compared to the other treatments, particularly with respect to the control sample.

Two papers reported the results of treatments with melatonin (*N*-acetyl-5-methoxytryptamine) applied to preserve and extend the shelf life of mangos and zucchinis. In the first study, a trial measuring the response of on two mango (*Mangifera indica* L.) cultivars to exogenous melatonin (1000 mol L^−1^) was conducted by Njie et al. [4]. During storage for about one month at 13 °C, physiological parameters, metabolic processes, and relative gene expression levels were measured. For both the cultivars analyzed (Guiqi and Tainong 1), the authors reported that the melatonin treatment did not affect the content of total soluble solids, titratable acidity, or the TSS:TA ratio; instead, it delayed weight loss, increased the period of firmness, lowered respiration rates, and reduced the incidence of decay. Furthermore, the melatonin treatment inhibited the decrease in ascorbic acid, flavonoid, and total phenol content as well as the enzyme activity of polyphenol oxidase. Additionally, for both mango cultivars, a delay in the period during which the malondialdehyde content increased during storage was observed. For both cultivars, in the fruit treated with melatonin, increases in the activity of antioxidant enzymes (superoxide dismutase and ascorbate peroxidase as wells as phenylalanine ammonia-lyase) and their genes’ relative expression were noticed compared to the untreated fruit.

Medina-Santamarina et al. [5] reported the efficacy of exogenous melatonin supplementation (1 mM) alone or in combination with 1-methylcyclopropene (1-MCP) 2400 ppbL^−1^ for reducing chilling damage in zucchinis (*Cucurbita pepo* spp. *pepo* cv. Cronos). Zucchinis were stored at 4 °C for 15 d and an additional 2 d at 20 °C. For the zucchinis treated with melatonin + 1 MCP, weight loss and signs of chilling damage were reduced compared to the other treatments and the control, maintaining a higher degree of fruit firmness throughout cold storage. Moreover, for each sampling day, the combined treatment (1-MCP + melatonin) effected the lowest values of malondialdehyde and electrolyte leakage. The combined treatment only reduced the respiration rate for 9 d, while ethylene production was reduced only by the treatment with 1-MCP alone.

While stored at 4 °C, the total soluble solids and total chlorophyll content in the combined treatment were comparable with 1-MCP application. The authors highlighted that the proposed combined treatment could be a promising tool for increasing the storability of zucchinis at suboptimal temperatures.

In another study, to retard peach senescence (*Prunus persica* L. cv. Xiahui 8), treatment with 1-MCP, alone or in combination with nitric oxide, was proposed [6]. At the end of the storage period (8 d at 25 °C), the proposed combined treatment (1-MCP + nitric oxide) yielded the best effects on fruit quality, maintaining good physical characteristics and decelerating the rate at which the fruit firmed, its respiration rate, and ethylene production. During storage, antioxidant enzymes (ROS inhibiting), which affect the ripening and senescence of fruit, were examined. Furthermore, for each compared treatment, twenty phenols were identified and quantified.

The research conducted by Mudalal et al. [7] revealed the preservation effects of different ingredients (fresh onion (*Allium cepa* L.), corn oil, salt, sumac (*Rhus coriaria* L.), and lactic acid) on the quality traits of fresh Za’atar (*Origanum syriacum*) sealed in vacuum bags and stored at 2–4 °C for 42 d. Microbial counts, color, and other sensory attributes were analyzed every 7 d. The study showed that the addition of lactic acid induced a strong preservative effect against aerobic and anaerobic bacteria. Moreover, the addition of sumac improved the preservation of vacuum-packaged oregano stored in refrigerated conditions.

Regarding the second topic, (ii) packaging and edible coating, Vieira et al. [8] reported the results of research on fresh red raspberries (*Rubus idaeus* L. cv. ‘Kweli’). To extend the shelf life of this perishable fruit, pads produced with chitosan, green tea, or rosemary ethanolic extracts were incorporated as natural antifungal agents. Sealed fruit trays were packaged under an air atmosphere using polyacid lactic film and stored at 4 °C for 14 d. At the end of the storage period, reductions in the growth and decay of spoiled fruit of 5% and 13% were observed for chitonsan + rosemery and chitonsan + green tea, respectively. Conversely, for the fruit packets without pads, these percentages were equal to 40 and 80% after 7 and 14 d, respectively. The use of polyacid lactic film reduces weight loss, thus preserving the firmness of raspberries, particularly when using pads with green tea or rosemary ethanolic extracts. Total phenols and ascorbic acid content were better preserved in the packages with film pads containing one of each extract. The results highlighted how the application of chitosan pads + rosemary ethanolic extracts could be applied to other soft fruit to increase their marketability.

As an edible coating, chitosan was also used to preserve the quality attributes of fresh date (*Phoenix dactylifera* L. cv Barhi) fruit in combination with extracts of olive cake and orange peel in two different concentrations (1 or 2%) during storage at 4 °C for 28 d [9]. When the chitosan was combined with either of the two extracts, a pronounced effect was observed. In particular, the treatment with olive cake (at 2%) increased the total phenolic content by five-fold and preserved DPPH inhibition to a three-fold greater degree compared to the control at the end of the storage period. For all the tested coatings, an increase in total soluble solids was observed, and the effects on moisture prevention, loss of firmness, and fungal growth did not affect sensory characteristics.

Imeneo et al. [10] studied the effect of an edible pectin-based coating integrated with a lemon extract by-product on the quality attributes of minimally processed carrots (*Daucus carota* cv. Nantes) stored at 4 °C for 14 d. The application of lemon extract preserved the fresh-cut carrots’ physiological parameters, thereby delaying their senescence. The carrots showed limited changes in color and white-blush and a good degree of firmness throughout storage due to the presence of calcium chloride in the coating’s formulation. The lemon extract by-product improved the microbiological stability of the minimally processed carrots, showing the lowest value of total bacterial activity after 7 d, while a mild increase was observed following the end of the storage period. Total carotenoids, phenolic content, and antioxidant activity values exhibited a similar trend during storage for all treatments, while higher levels were measured in the fresh-cut carrots treated with lemon extract.

Regarding topic (iii) concerning the techniques or markers for determining fruit quality, two papers focused their activity on Candonga strawberries (*Fragaria x ananassa* Duch. cv Sabrosa). The first one, proposed by Cozzolino et al. [11], focused on studying the profiles of volatile and phenolic compounds via HPLC as markers of the strawberries’ ripening stage. Strawberries picked at three different harvest times and two ripening stages, namely, when half-red and red, were evaluated. An analysis of polyphenolic compounds revealed that the concentration of anthocyanins increased during the harvest times, whereas the content of flavonoids declined. Overall, fifty-seven volatile compounds were identified, including esters, aldehydes, alcohols, acids, terpenes, furanones, lactones, and others. A multivariate analysis was carried out, for which all the chemical data were considered. The results highlighted that the fruit at the red ripening stage during the first and second harvesting periods were similar, while the red fruit of the third harvesting stage significantly differed. This difference likely indicated overripening or an early stage of fruit senescence. Finally, the authors identified butyl butyrate, ethyl hexanoate, hexyl acetate, nonanal, terpenes, and lactones represented the volatile organic compounds that had a positive impact on consumers’ preferences.

The objective of the research conducted by Palumbo et al. [12] was to discriminate two ripening stages (red and half-red) of strawberries, which were harvested at different times during the harvest season, by comparing different techniques, namely, image analysis, the use of an electronic nose, and attenuated total reflection-Fourier transform infrared (ATR-FTIR) spectroscopy. In the principal results concerning a correlation analysis between the data obtained via the e-nose and an analysis of volatile organic compounds, it was revealed that the HS-SPME/MS e-nose experimental data contained a sufficient amount information with which to allow for the discrimination of strawberry samples based on their stage of ripening. Moreover, titratable acidity was correlated with ATR-FTIR and image analysis data. Since titratable acidity usually decreases in strawberries during ripening, its assessment by ATR-FTIR or image analysis might provide a suitable indicator for a fast and non-destructive evaluation of the ripening stages of this fruit.

Hamie et al. [13] studied the possibility of using a non-destructive tool to determine the maturity stage of table grapes (*Vitis vinifera* L. cv. Sugranineteen) during the ripening season. Multiplex^®^ 3 (FORCE–A, Orsay, France) equipped with a portable optical sensor was used for analysis. The study focused on the measurement of skin anthocyanin using two fluorescence indexes, namely, ANTH_RG (chlorophyll fluorescence excited with red and green lights) and FERARI (fluorescence excitation ratio anthocyanin relative index), and other indices concerning the total flavonoid, nitrogen, and chlorophyll content. All measurements were correlated with the principal quality parameters (color, pH, total soluble solids, titratable acidity, total phenols, antioxidant activity, anthocyanin, and flavonoids) obtained by applying common analysis techniques. The results highlighted an important relationship between the total anthocyanins (measured via spectrophotometry) and ANTH_RG and FERARI indices with R^2^ values equal to 0.96 and 0.87, respectively. The main result obtained by the researchers was a regression equation developed using the ANTH_RG index (measured by Multiplex^®^ 3) and skin anthocyanin content (obtained via common analysis techniques), which allows one to estimate the previously mentioned parameters directly in-vineyard in rapid mode and without damaging the plant material.

Owoyemi et al. conducted phenotyping analysis of the effects of various preharvest and postharvest quality levels of navel oranges (*Citrus sinensis* L. cv Rustenburg) in order to develop shelf-life prediction models with which to enable the use of a First Expired–First Out (FEFO) logistics strategy [14]. During the analysis, fruit originating from six different orchards was stored for 20 weeks at different temperatures and relative humidities (at 5 °C with 70, 90, or 95% relative humidity or at 2 or 8 °C with 90% relative humidity). Fourteen fruit quality parameters were analyzed every week for each cold storage condition and after one-week of shelf storage at ambient temperature (20 °C). Four different linear and non-linear regression models were tested to determine their ability to predict fruit acceptance scores, namely, multiple linear regression, support vector regression, random forest, and extreme gradient boosting. Based on the obtained data, among the different regression models, extreme gradient boosting combined with a duplication approach provided the most effective approach to predicting fruit quality, yielding an RMSE of 0.217 and an R^2^ of 0.891. Thus, in the future, the development of accurate shelf-life prediction models should contribute to optimizing the FEFO logistic management system and thus promote more efficient inventory management and loss reduction. Postharvest storage and preservation strategies for extending the storage life of the king oyster mushroom (*Pleurotus eryngii* D.C.) were described in the review article written by Gou et al. [15]. Many storage preservation techniques, including physical and chemical methods, were described. A suitable storage atmosphere, appropriate packaging, and optimal storage conditions (in terms of temperature and relative humidity) as well as irradiation or drying were some of the preservation methods described in the paper. Other strategies described for preserving the shelf-life of *Pleurotus eryngii* were the use of the lactic acid fermentation process and nanoparticles with which to encapsulate bioactive substances. According to Gou et al. [15], future research on *Pleurotus eryngii* should focus on combining new and traditional technologies to improve postharvest quality. Among such techniques, it was stressed that radiation treatment with 1-MCP alongside a nano-packaging treatment, safe and efficient sterilization, microwaving, and the use of low-pressure electrostatic fields and low-temperature plasma sterilization equipment could be successfully applied during the storage and distribution of king oyster mushrooms. In the study conducted by Palumbo et al. [16], the latest postharvest technologies capable of extending the shelf-life of fruit and vegetables were described. Physical treatments such as microwave heating and the application of high hydrostatic pressure, pulsed electric fields, and cold plasma were reported to reduce microbial load and preserve the freshness and quality characteristics of fruits and vegetables. Moreover, a section about dipping and vacuum impregnation treatment as well as edible active packaging based on natural compounds (including alginate, chitosan, lemon by-product or essential oil, orange peel, and olive cake) was presented. One paragraph described the opportunities offered by selected microbes as control agents since biocontrol is considered one of the more sustainable postharvest approaches for increasing the shelf-life of whole and fresh-cut fruits and vegetables. Examples of innovative, non-destructive techniques for the quality monitoring of fruits and vegetables were described. Among these, it was revealed that image analysis based on traditional imaging in the visible range of the electromagnetic spectrum using a computer vision system is widely used for the in-line grading of many types of fruits and vegetables. Furthermore, it was related that the electronic nose has become one of the most favorable sensing technologies for evaluating the presence and content of specific volatile metabolites corresponding to the presence or loss of the freshness of fresh vegetable products. Finally, near-infrared spectroscopy was described as a technique that can be used to analyze the chemical composition of fresh produce and quality changes during storage. The authors report that this novel technology, which can be applied directly on fields or in industrial lines, is a valid approach for ensuring the traceability and authentication of agricultural produce.

In conclusion, the papers proposed in this Special Issue, which were written within different European and national projects or promoted by private enterprises, report innovative results that extend our knowledge of the postharvest preservation technology applied to fruit and vegetables that are either fresh or minimally processed. The Guest Editors thank all authors that have contributed to enriching the Special Issue and hope that the results presented might constitute a stimulus for further studies.
